# Assessing repeatability and reproducibility of Anterior Active Rhinomanometry (AAR) in children

**DOI:** 10.1186/s12874-020-00969-1

**Published:** 2020-04-17

**Authors:** Giovanna Cilluffo, Anna Maria Zicari, Giuliana Ferrante, Velia Malizia, Salvatore Fasola, Marzia Duse, Giovanna De Castro, Valentina De Vittori, Laura Schiavi, Giulia Brindisi, Paolo Petrelli, Stefania La Grutta

**Affiliations:** 1 Institute for Biomedical Research and Innovation (IRIB), National Research Council (CNR), Via Ugo La Malfa 153, Palermo, Italy; 2 Department of Pediatrics, Allergology and Immunology Division, Sapienza University, Viale Regina Elena 324, Rome, Italy; 3 Department of Health Promotion Sciences, Maternal and Infant Care, Internal Medicine and Medical Specialities “G. D’Alessandro”, University of Palermo, Viale delle scienze Ed. 13, Palermo, Italy; 4 Medi-Care Solutions S.r.l. Euroclinic®, Via Pietro Nenni 3, Imola (BO), Italy

**Keywords:** Anterior Active Rhinomanometry, Children, Coefficient of Variation, Intraclass Correlation Coefficient, Rhinitis, Repeatability

## Abstract

**Background:**

Repeatability and reproducibility are essential for clinicians for several purposes. Although discouraged, use of the Coefficient of Variation (CV) for assessing repeatability and reproducibility, rather than the Intraclass Correlation Coefficient (ICC), is still widespread. The aim of the present study was to highlight how using inappropriate indices may lead to misleading results, and this is done by simulation study and using real data on Anterior Active Rhinomanometry (AAR) in both healthy children and ones with rhinitis.

**Methods:**

A simulation study was carried out to highlight how using inappropriate indices could be misleading. Then a comparison was made between CV and ICC to assess repeatability and reproducibility of AAR, for which previous studies have given underestimated results. AAR is recommended as the gold standard tool for measuring nasal resistance in clinical practice.

**Results:**

A simulation study showed that the ICCs estimated from data generated assuming a true CV yielded results in agreement with estimated CVs; by contrast, if data were generated assuming a true ICC, CVs yielded conflicting results. For AAR, ICCs showed good repeatability, whereas CVs showed unacceptable repeatability. AUC and 95% CI for AAR showed good performance in predicting current symptoms of rhinitis in the overall study population.

**Conclusions:**

The present study focused on the importance of the choice of appropriate indices of repeatability and reproducibility, demonstrating the repeatability of AAR in both healthy children and ones with rhinitis.

**Trial registration:**

ClinicalTrials.gov (ID: NCT03286049; Registration Date: September 15, 2017; Actual Study Start Date: January 10, 2018).

## Background

Repeatability of measurements refers to the variation in repeated measurements made on the same subject under identical conditions. Variability in measurements made on the same subject in a repeatability study can then be ascribed only to errors due to the measurement process itself [1]. By contrast, when the measurements are performed under changing conditions, i.e. over a period of time, reproducibility is assessed. Repeatability and reproducibility are essential for clinicians for a variety of purposes [2, 3], such as aiding diagnosis, predicting future patient outcomes and choosing a personalized therapy. Several statistical methods have been developed and recommended for assessing repeatability and reproducibility, i.e. Intraclass Correlation Coefficient (ICC) and Bland Altman plot, whereas others have been discouraged, for example Pearson’s correlation and Coefficient of Variation (CV) [1, 4, 5].

This paper was motivated by a study on Anterior Active Rhinomanometry (AAR) in healthy children and in ones with rhinitis. AAR is recommended as the gold standard tool for measuring nasal ventilation during a normal respiratory cycle and resistance at the nostrils in patients with upper airway obstruction symptoms [5, 6]. In clinical practice, AAR is the most widely used and readily applicable test for assessing the degree of nasal obstruction, as well as for monitoring clinical outcomes after surgical or medical procedures in order to improve nasal patency [7]. The test execution procedure is standardized according to the International Committee on Standardization of Rhinomanometry [6], with subjects sitting in upright positon and wearing a face mask, where breathe only with the nose and close their mouth.

To date, few studies investigating AAR repeatability have been performed in adults only, showing controversial results [8–10]. In particular, Carney et al. observed that single measurements had an unacceptably high CV (19–60%) in a cross-sectional study on seven adults [9], and Thulesius et al. reported rather poor long-term reproducibility (CV 27%) in a longitudinal study over 5 months on nine healthy adults [10]. Conversely, Silkoff et al. reported a high level of repeatability (coefficient of variation, CV 8.5 ± 2.8%) and Intraclass Correlation Coefficient (ICC) 0.96 in a small sample of healthy subjects [8].

The aim of the present study was to highlight the fact that using inappropriate tools may lead to misleading results, and this was done by comparing the ICC, the Bland Altman plot and the CV for data from both healthy children and ones with rhinitis and by a simulation study, as a possible reference for clinicians dealing with this type of study.

## Methods

### Statistical tools and underlying assumptions

This section is devoted to introducing the statistical tools used in the simulation and clinical data. The ICC can be defined as the ratio of the between-subject variance to the sum of the within-subject and between-subject variances, and can be derived from a two-level random effect model [11]:

$$ ICC=\frac{\sigma_B^2}{\sigma_B^2+{\sigma}_W^2} $$

The ICC ranges from 0 to 1 and the following benchmarks can be used for interpretation: ICC < 0.20 “poor agreement”, 0.21–0.40 “fair agreement”, 0.41–0.60 “moderate agreement”, 0.61–0.80 “substantial agreement”, and > 0.80 “excellent agreement” [12–14]. In order to detect at least “fair agreement”, a significance test [15] can be performed to assess the following hypotheses:

$$ \left\{\begin{array}{c}{H}_0: ICC\le 0.20\\ {}{H}_1: ICC>0.20\end{array}\right. $$

The ICC suffers from a variety of methodological issues including sensitivity to assumptions of normality and equal variance [16, 17], and its use under assumption violations leads to misleading and likely inflated estimates of interrater reliability [18].

The CV is defined as the ratio between the standard deviation and the mean:

$$ {CV}_i=\frac{\sigma_i}{\mu_i} $$

where *σ*_*i*_ and *μ*_*i*_ are, respectively, the standard deviation and the mean of the measurement for subject *i*. CV is subject to some restrictions; for example it is meaningful only for measurements with a real zero (i.e., “ratio scales”). In addition, the values of the measurement to compute the CV always have to be positive [19]. The levels of acceptability for the CV depend on the field of application [20, 21]; however, CV < 15% is widely used [9, 10].

The Bland-Altman plot is used to assess the agreement between two repeated measurements [22] and to visually check possible heteroscedasticity of the data. Heteroscedasticity means that the size of the difference between two measurements changes with the size of the mean of the two measurements. Logarithmic transformation is suggested in the case of heteroscedasticity [23]. A nonparametric approach is recommended when the paired differences are not normally distributed [24].

### Simulation study

The simulation scenarios were inspired by our real data. We simulated data assuming two different generating mechanisms. In the first batch of simulations, we generated 1000 replicates from a normal distribution with a fixed CV, hypothesizing *n* = 10 subjects each with *p* = 5 repeated measurements. In particular, for each subject the p measurements were generated from X_*i*_ ∼ *N*(*μ*_*i*_, *σ*_*i*_), with *μ*_*i*_ ranging from 5 to 8 (10 equally spaced values), and *σ*_*i*_ = *μ*_*i*_∗CV, with CV ranging from 0.01 to 0.99 (50 equally spaced values). At each replication, the ICC was estimated.

In the second batch of simulations, we generated 1000 replicates for *n* = 10 subjects each with *p* = 5 repeated measurements from a mixed model. In particular, for each subject the p measurements were generated from ${\mathrm{X}}_i\sim {N}_p\left({\mu}_i,{\sigma}_B^2\right)$, with ${\mu}_i\sim N\left({\gamma}_i,{\sigma}_B^2\right)$. Different configurations were considered by varying the overall mean *γ*_*i*_ = 1, 2…10, the between-subject variance ${\sigma}_B^2=1,4,9$, and within-variances ${\sigma}_W^2$ varied, for fixed ${\sigma}_B^2$, to simulate a true ICC sequence from 0.10 to 0.90 (9 equally spaced values). At each replication, the CV was estimated.

### Clinical data

The data analysed in the present paper arise from a multicentre observational study carried out at the Pediatric Allergy and Immunology Service, Sapienza University (Rome, Italy), and at the Pulmonary and Allergy Pediatric Clinic of the CNR-IBIM (Palermo, Italy). The study was approved by the local Institutional Ethics Committee (Palermo, Italy, Approval Number: 7/2017), and informed consent was obtained from all parents before study entry. Once approved, the study was registered on ClinicalTrials.gov (ID: NCT03286049). This study was conducted in accordance with Good Clinical Practice and the Declaration of Helsinki.

The sample size was estimated according to the method illustrated by Zou [25] using the *ICC.Sample.Size* R package [26]. In order to test the null hypothesis of ICC ≤ 0.20, considering an expected ICC of 0.70 based on a previous study [8], five repeated measurements per subject and a 90% statistical power and a 5% significance level, a sample size of 10 subjects per group was required. Therefore, the study population comprised 50 children, i.e. 10 subjects for each of the following 5 groups:

- Healthy Children (HC)
- Children with non-allergic rhinitis (NAR), i.e. children with rhinitis symptoms but without allergic sensitization;
- Children with perennial allergic rhinitis (PAR), i.e. children sensitized to perennial allergens;
- Children with seasonal allergic rhinitis outside (SAR-O) and during (SAR-D) the pollen season, i.e. children sensitized to seasonal allergens;

All the children underwent a standardized questionnaire including demographic characteristics and the core questions on rhinitis of the International Study on Childhood Asthma and Allergy (ISAAC) [27]. The questions referred to problems with sneezing, or a runny, or blocked nose when the child did not have a cold or the ‘flu, “ever” and “in the past twelve months”.

The inclusion criteria were the following: (*1*) age 10–16 years; (*2*) Total Five Symptoms Score (T5SS) > 5 for children with AR and NAR; the T5SS included sneezing, rhinorrhea, nasal itching, nasal obstruction and itchy eyes (each symptom score ranging from 0 –absent- to 3 –severe-, so that the maximum possible score was 15); T5SS > 5 at inclusion was established to ensure that patients were symptomatic. The exclusion criteria were the following: medical diagnosis of nasal anatomic defects (i.e., deviated septum) or nasal polyp disease; craniofacial malformations; genetic diseases; medical diagnosis of asthma according to GINA guidelines (http://ginasthma.org); any acute illness in progress and in the month before the study; use of systemic steroids or antihistamines in the past 4 weeks; use of any nasal therapy in the past 4 weeks; active smoking. The study involved three visits: screening (visit 1, baseline), visit 2 (after 14 ± 3 days), and a final assessment (visit 3, after 28 ± 3 days). At visit 1, patients were assessed for eligibility and recruited if they met the inclusion criteria; then they underwent physical examination and five AAR measurements for each nostril. At visit 2 and 3, patients underwent one AAR measure for each nostril. The performance of AAR parameters in predicting patients’ current symptoms of rhinitis was assessed through a ROC analysis [28]. The estimation of the Area Under the Curve (AUC) was performed by nonparametric ROC analysis and significance was tested using the method described by DeLong et al. [29]. Moreover, to avoid overrating the test performance in ROC analysis, we performed a five-fold cross validation [30]. A *p*-value < 0.05 was considered to indicate a statistically significant effect. Statistical analyses were performed through R version 3.5.2; ICCs were computed using the R package *irr* [15], the ROC curves were computed using pROC [31].

### Anterior active Rhinomanometry (AAR)

AAR was performed according to the ICSR guidelines, using a RINOPOCKET ED200 (EUROCLINIC®, ITALY) rhinomanometer. The rhinomanometer was calibrated according to standard requirements. Rhinomanometry was done in a temperature- and humidity-controlled room. A small plastic catheter was inserted through a pierced piece of tape and attached to flexible silicone tubing leading to the pressure port of the meter. The foam was placed across the contralateral nostril to measure the nasal pharyngeal pressure, taking care not to interfere with the nostril being tested. The tubing was brought out around the side of the transparent mask. To perform rhinomanometry patients were asked to wear a face mask, close their mouths and breathe. For each nostril a rhinogram was recorded which related inspiratory and expiratory nasal airflow to transnasal pressure. A retest was performed in all patients. Measurements were performed by the same operator using the same instrument and following the standard operation procedure according to Clement [32].

In reference to Ohm’s law (R = DeltaP / F), Rinopocket uses the following: 1) a differential pressure transducer − 25 to + 25 KPa (− 3.6 to + 3.6 psi) temperature compensated to get DeltaP {other features are: accuracy (0 to 85 °C) = ±5.0%VFSS; sensitivity (V/P) = Typ 90 mV/KPa; response time (t _r_) = Typ 1.0 ms; offset stability = Typ ±0.5%VFSS}; 2) an airflow sensor compensated and amplified (±300 SLPM) to get Flow; {other features are: repeatability and hysteresis = Typ ±0.035 Vdc; response time (t _r_) = Typ 10 ms; Null voltage shift (25 °C to 5 °C [77 °F to 41 °F] = Typ ±0.02 Vdc; 25 °C to 60 °C [77 °F to 140 °F]) = Typ ±0.02 Vdc; full scale output shift (25 °C to 5 °C [77 °F to 41 °F] = Typ ±2.5%reading; 25 °C to 60 °C [77 °F to 140 °F]) = Typ ±2.5%reading}; 3) CPU = STM32F373 32bit with internal A/D converter (3CH 16bit sigma-delta); 4) EDM software to calculate AAR resistances at 150, 100, and 75 Pa (R 150 Pa, R 100 Pa and R 75 Pa), total resistance and other parameters such as max press, max flux, flux at 150,100, and 75 Pa. According to Broms, the quotient pressure-flow at the standardized points were the curves cross the circle with radius 2 which defined resistance 2 (R2) [33]. For each nasal resistance, the AAR parameters considered were inspiratory (R, L and R + L), expiratory (R, L and R + L), total combined (total inspiratory + total expiratory).

## Results

### Simulation study

**Fig. 1:**
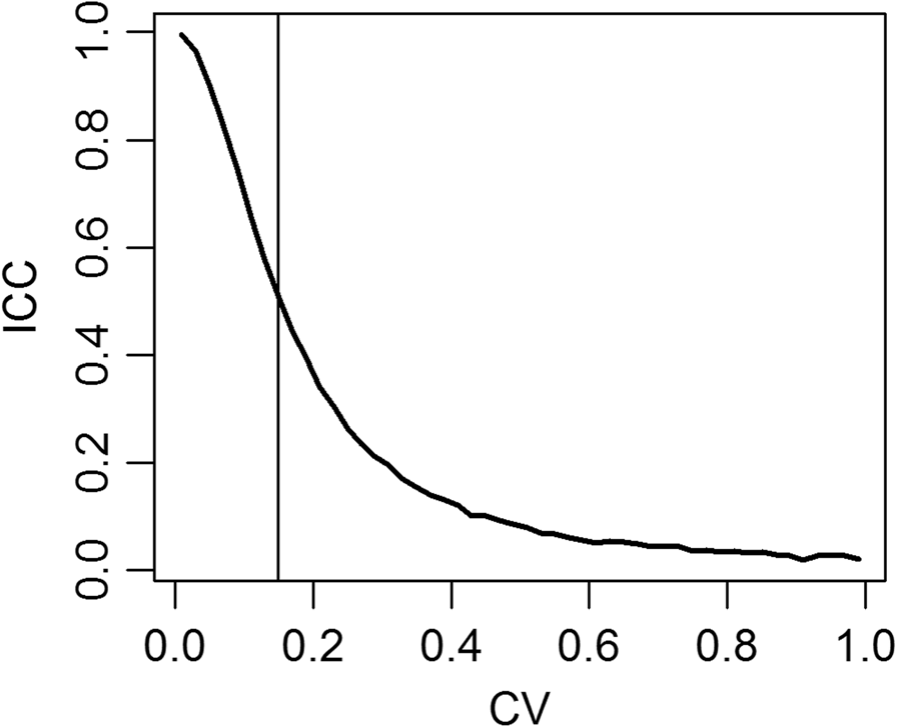
Simulated mean of the ICCs estimated given the CVs

Figure 1 shows the mean of the ICCs estimated given the CVs. The first batch of simulations emphasizes that until the true CV was < 15%, ICC was greater than 0.50 even if data were generated under the CV model; overall, ICC decreased as CV increased.

Table 1 reports the CVs estimated in the second batch of simulations. For fixed ICC (for fixed *σ*_*W*_ and *σ*_*B*_), the estimated CVs decreased as the overall mean μ increased as expected; however, most of the CVs were ≥ 0.15 also for high ICC values. For fixed μ, the estimated CV decreased as *σ*_*W*_ decreased as expected; the only CVs < 0.15 were observed for quite large *μ* values.

**Table 1:** Simulated means of the CVs with *n* = 10 and *p* = 5, for different σ_B,_ σ_W_ and overall mean μ

σ_B_	σ_W_	ICC	μ = 1	μ = 2	μ = 3	μ = 4	μ = 5	μ = 6	μ = 7	μ = 8	μ = 9	μ = 10
1	3.00	0.10	10.043	3.748	1.367	0.829	0.642	0.531	0.447	0.39	0.349	0.311
	2.00	0.20	8.384	1.517	0.773	0.563	0.442	0.367	0.316	0.274	0.243	0.220
	1.53	0.30	5.449	1.033	0.613	0.455	0.360	0.300	0.256	0.223	0.198	0.179
	1.22	0.40	7.652	0.844	0.527	0.392	0.311	0.257	0.221	0.193	0.172	0.154
	1.00	0.50	4.553	0.74	0.467	0.35	0.277	0.230	0.197	0.172	0.153	0.138
	0.82	0.60	2.237	0.661	0.422	0.316	0.254	0.209	0.179	0.158	0.14	0.126
	0.65	0.70	1.548	0.607	0.393	0.294	0.234	0.194	0.166	0.147	0.129	0.117
	0.50	0.80	1.372	0.566	0.365	0.274	0.218	0.181	0.156	0.137	0.121	0.109
	0.33	0.90	1.381	0.53	0.344	0.258	0.206	0.171	0.146	0.129	0.114	0.103
2	6.00	0.10	15.189	31.267	7.798	4.253	1.897	2.664	0.999	0.831	0.734	0.645
	4.00	0.20	39.446	8.42	4.951	1.589	0.971	0.774	0.654	0.562	0.496	0.448
	3.06	0.30	17.292	4.903	42.938	1.016	0.761	0.618	0.524	0.454	0.401	0.363
	2.45	0.40	14.228	3.779	1.431	0.835	0.643	0.525	0.449	0.392	0.346	0.312
	2.00	0.50	11.276	3.226	1.008	0.741	0.571	0.468	0.399	0.348	0.309	0.278
	1.63	0.60	7.749	2.987	0.908	0.654	0.52	0.423	0.362	0.319	0.281	0.254
	1.31	0.70	8.677	1.681	0.83	0.605	0.478	0.392	0.334	0.295	0.260	0.235
	1.00	0.80	6.518	1.544	0.761	0.562	0.445	0.366	0.314	0.276	0.244	0.219
	0.67	0.90	8.934	1.292	0.713	0.527	0.419	0.344	0.295	0.260	0.230	0.207
3	9.00	0.10	20.852	17.435	12.41	10.566	24.537	3.814	2.238	2.669	1.740	1.075
	6.00	0.20	17.845	15.271	8.064	4.595	2.732	1.501	1.089	0.891	0.774	0.695
	4.58	0.30	22.043	11.754	5.801	2.702	1.561	0.994	0.825	0.704	0.616	0.556
	3.67	0.40	16.047	9.100	5.331	1.661	1.052	0.829	0.694	0.603	0.527	0.476
	3.00	0.50	55.490	6.752	3.387	1.563	0.911	0.726	0.612	0.531	0.470	0.422
	2.45	0.60	147.388	6.271	1.847	1.155	0.819	0.651	0.554	0.486	0.426	0.385
	1.96	0.70	13.316	5.836	1.746	1.058	0.744	0.600	0.509	0.447	0.393	0.355
	1.50	0.80	12.635	7.708	1.421	0.927	0.689	0.559	0.478	0.418	0.369	0.332
	1.00	0.90	10.619	4.621	1.207	0.841	0.647	0.524	0.447	0.394	0.347	0.312

### Repeatability of AAR

At baseline, the characteristics of the children were similar in the five groups (Table 2). In Table 3 the AAR parameters given the five groups are shown. Significant differences were found for all AAR parameters among groups. Table 4 reports the within-day ICCs for each AAR parameter by group. Most of the ICCs were statistically significant in all groups and they were > 0.20, which is considered the cut-off value between poor and fair agreement. Table 5 reports the coefficient of variation by group for all AAR. Most of the CVs were ≥ 0.15, which would indicate unacceptable repeatability.

**Table 2:** Characteristics of children by group at the baseline visit

	HC	NAR	PAR	SAR-O	SAR-D	All	*p*-value*
n	10	10	10	10	10	50	
Female	6 (60.00)	5 (50.00)	4 (40.00)	4 (40.00)	5 (50.00)	24 (48.00)	0.891
Age, *years*	11.30 ± 1.64	12.20 ± 1.14	12.00 ± 2.31	11.30 ± 1.49	12.00 ± 1.70	11.76 ± 1.67	0.486
Weight, *Kg*	56.20 ± 28.67	52.10 ± 11.72	50.90 ± 12.71	44.90 ± 7.48	44.90 ± 7.94	49.80 ± 15.72	0.490
Height, *cm*	155.15 ± 14.63	155.90 ± 9.62	154.50 ± 15.44	146.40 ± 7.90	151.45 ± 10.87	152.68 ± 12.07	0.375
BMI, *Kg/m*^*2*^	22.27 ± 6.34	21.22 ± 3.33	21.07 ± 2.70	20.83 ± 1.95	19.43 ± 1.11	20.97 ± 3.54	0.325
Parental history of rhinitis	4 (40.00)	4 (40.00)	6 (60.00)	5 (50.00)	7 (70.00)	26 (52.00)	0.605
Parental history of asthma	1 (10.00)	0 (0.00)	2 (20.00)	0 (0.00)	1 (10.00)	4 (8.00)	0.433
Parental smoking exposure^#^	3 (30.00)	5 (50.00)	2 (20.00)	0 (0.00)	3 (30.00)	13 (26.00)	0.143
Current symptoms of rhinitis**	6 (60.00)	8 (80.00)	9 (90.00)	10 (100.00)	10 (100.00)	43 (86.00)	0.054

Data are presented as mean ± SD for quantitative variables, n (%) for categorical variables. ^*^Kruskal-Wallis test for quantitative variables, χ^2^ test for categorical variables. ^#^last 12 months; **ISAAC core question for rhinitis, n. 2: “In the past 12 months, has your child had a problem with sneezing, or a runny nose, or blocked nose when he/she did not have a cold or the flu? Yes/No″

**Table 3:** Nasal resistances (R2, R 75 Pa, R 100 Pa, R 150 Pa) by group

	HC	NAR	PAR	SAR-O	SAR-D	*p*-value
n	10	10	10	10	10	
**R2**						
Total Inspiratory	1.17 ± 0.52	1.18 ± 0.66	1.91 ± 1.69	2.56 ± 5.16	8.85 ± 11.77	**0.024**
Tota Expiratory	1.06 ± 0.50	1.20 ± 0.71	1.82 ± 1.52	1.86 ± 3.18	8.39 ± 10.69	**0.010**
Combined Total	2.23 ± 1.01	2.38 ± 1.32	3.74 ± 3.21	4.43 ± 8.33	17.24 ± 22.45	**0.016**
**R75 Pa**						
Total Inspiratory	1.09 ± 0.47	1.06 ± 0.66	1.85 ± 1.53	2.20 ± 2.40	8.48 ± 8.57	**< 0.001**
Tota Expiratory	0.93 ± 0.48	0.80 ± 0.88	1.85 ± 1.59	2.38 ± 2.41	8.87 ± 7.63	**< 0.001**
Combined Total	2.02 ± 0.94	1.86 ± 1.46	3.69 ± 3.12	4.58 ± 4.81	17.36 ± 16.09	**< 0.001**
**R 100 Pa**						
Total Inspiratory	1.09 ± 0.47	1.03 ± 0.66	1.80 ± 1.52	1.99 ± 2.43	7.98 ± 8.75	**0.002**
Tota Expiratory	0.93 ± 0.51	0.68 ± 0.84	1.56 ± 1.47	1.90 ± 1.80	6.41 ± 4.74	**< 0.001**
Combined Total	2.02 ± 0.96	1.71 ± 1.47	3.36 ± 2.97	3.89 ± 4.23	14.38 ± 12.85	**< 0.001**
**R 150 Pa**						
Total Inspiratory	1.10 ± 0.61	0.81 ± 0.80	1.88 ± 1.80	2.57 ± 5.15	8.88 ± 11.77	**0.019**
Tota Expiratory	0.64 ± 0.52	0.62 ± 0.89	1.47 ± 1.63	1.98 ± 3.28	8.41 ± 10.68	**0.006**
Combined Total	1.74 ± 1.06	1.43 ± 1.67	3.35 ± 3.36	4.55 ± 8.42	17.28 ± 22.43	**0.011**

**Table 4:** Within-day ICCs by group for all the measured nasal resistances (R2, R 75 Pa, R 100 Pa, R 150 Pa)

	Inspiratory						Expiratory						Combined Total	
	R		L		TOT		R		L		TOT			
R2	ICC	p-value	ICC	p-value	ICC	p-value	ICC	p-value	ICC	p-value	ICC	p-value	ICC	p-value
HC	0.514	**0.012**	0.527	**0.009**	0.336	0.158	0.561	**0.004**	0.452	**0.036**	0.389	0.088	0.37	0.11
NAR	0.511	**0.013**	0.53	**0.009**	0.541	**0.007**	0.388	**0.088**	0.348	0.14	0.394	0.081	0.524	**0.01**
PAR	0.833	**< 0.001**	0.845	**< 0.001**	0.863	**< 0.001**	0.846	**< 0.001**	0.832	**< 0.001**	0.866	**< 0.001**	0.867	**< 0.001**
SAR-O	0.745	**< 0.001**	0.954	**< 0.001**	0.748	**< 0.001**	0.881	**< 0.001**	0.946	**< 0.001**	0.885	**< 0.001**	0.809	**< 0.001**
SAR-D	0.737	**< 0.001**	0.787	**< 0.001**	0.727	**< 0.001**	0.69	**< 0.001**	0.794	**< 0.001**	0.677	**< 0.001**	0.705	**< 0.001**
**R 75 Pa**														
HC	0.469	**0.027**	0.432	**0.049**	0.241	0.352	0.47	**0.027**	0.55	**0.006**	0.303	0.216	0.271	0.282
NAR	0.36	0.123	0.619	**0.001**	0.502	**0.015**	0.787	**< 0.001**	0.54	**0.007**	0.68	**< 0.001**	0.67	**< 0.001**
PAR	0.953	**< 0.001**	0.838	**< 0.001**	0.928	**< 0.001**	0.962	**< 0.001**	0.783	**< 0.001**	0.914	**< 0.001**	0.926	**< 0.001**
SAR-O	0.802	**< 0.001**	0.784	**< 0.001**	0.802	**< 0.001**	0.816	**< 0.001**	0.718	**< 0.001**	0.776	**< 0.001**	0.79	**< 0.001**
SAR-D	0.866	**< 0.001**	0.399	0.077	0.817	**< 0.001**	0.756	**< 0.001**	0.836	**< 0.001**	0.726	**< 0.001**	0.782	**< 0.001**
**R 100 Pa**														
HC	0.469	**0.028**	0.52	**0.011**	0.264	0.298	0.472	**0.026**	0.529	**0.009**	0.314	0.196	0.292	0.239
NAR	0.274	0.276	0.659	**< 0.001**	0.448	**0.038**	0.811	**< 0.001**	0.608	**0.001**	0.731	**< 0.001**	0.645	**< 0.001**
PAR	0.79	**< 0.001**	0.671	**< 0.001**	0.939	**< 0.001**	0.79	**< 0.001**	0.843	**< 0.001**	0.889	**< 0.001**	0.937	**< 0.001**
SAR-O	0.420	**0.05**	0.186	0.493	0.811	**< 0.001**	0.415	0.068	0.682	**< 0.001**	0.409	0.068	0.64	**< 0.001**
SAR-D	0.336	0.158	0.195	0.469	0.858	**< 0.001**	0.324	0.179	0.714	**< 0.001**	0.431	**0.049**	0.766	**< 0.001**
**R 150 Pa**														
HC	0.513	**0.012**	0.591	**0.002**	0.383	0.094	0.464	**0.03**	0.247	0.339	0.337	0.156	0.363	0.118
NAR	0.561	**0.004**	0.655	**< 0.001**	0.667	**< 0.001**	0.834	**< 0.001**	0.623	**0.001**	0.768	**< 0.001**	0.738	**< 0.001**
PAR	0.828	**< 0.001**	0.841	**< 0.001**	0.879	**< 0.001**	0.859	**< 0.001**	0.568	**0.004**	0.777	**< 0.001**	0.851	**< 0.001**
SAR-O	0.743	**< 0.001**	0.952	**< 0.001**	0.747	**< 0.001**	0.883	**< 0.001**	0.713	**< 0.001**	0.884	**< 0.001**	0.816	**< 0.001**
SAR-D	0.739	**< 0.001**	0.785	**< 0.001**	0.727	**< 0.001**	0.687	**< 0.001**	0.807	**< 0.001**	0.674	**< 0.001**	0.703	**< 0.001**

Data are presented as ICC. Significant *p*-values are shown in bold

**Table 5:** Within-day CV by group for all the measured nasal resistances (R2, R 75 Pa, R 100 Pa, R 150 Pa)

	Inspiratory			Expiratory			Combined Total
	R	L	Tot	R	L	Tot	
**R2**							
HC	0.506	0.37	0.393	0.563	0.359	0.402	0.385
NAR	0.515	0.529	0.394	0.678	0.634	0.425	0.380
PAR	0.375	0.354	0.287	0.449	0.324	0.297	0.280
SAR-O	0.183	0.213	0.153	0.167	0.234	0.148	0.150
SAR-D	0.271	0.324	0.239	0.286	0.353	0.26	0.244
**R 75 Pa**							
HC	0.710	0.449	0.47	0.811	0.994	0.591	0.500
NAR	0.736	0.630	0.586	0.923	0.803	0.708	0.613
PAR	0.454	0.419	0.309	0.77	0.627	0.471	0.354
SAR-O	0.389	0.359	0.313	0.271	0.321	0.205	0.239
SAR-D	0.270	0.402	0.341	0.301	0.274	0.285	0.293
**R 100 Pa**							
HC	0.666	0.690	0.457	0.932	0.931	0.717	0.513
NAR	1.036	0.532	0.622	0.804	0.922	0.766	0.667
PAR	0.714	0.656	0.382	0.68	0.624	0.437	0.365
SAR-O	0.357	0.393	0.218	0.362	0.381	0.244	0.180
SAR-D	0.333	0.437	0.224	0.356	0.287	0.283	0.220
**R 150 Pa**							
HC	0.591	0.557	0.489	1.414	1.330	1.004	0.584
NAR	1.191	0.968	1.032	0.775	1.390	0.818	1.063
PAR	0.789	0.569	0.599	0.461	0.686	0.654	0.537
SAR-O	0.189	0.217	0.159	0.307	0.521	0.355	0.260
SAR-D	0.264	0.327	0.243	0.315	0.350	0.270	0.250

### Reproducibility of AAR

**Fig. 2:**
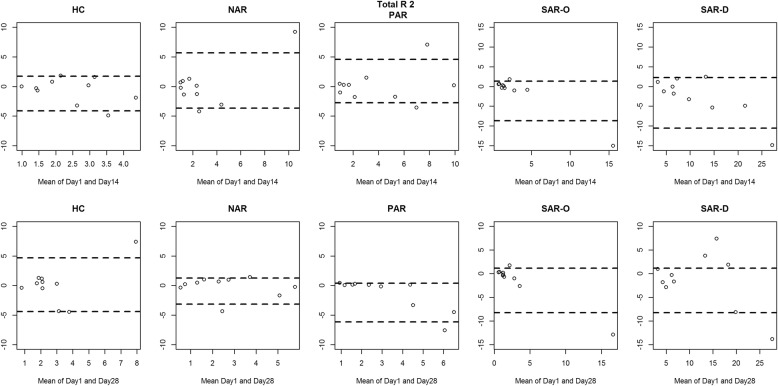
Bland-Altman plot: the difference between the Total R2 measurements of Day 1 and Day 14 (first row) and between Day 1 and Day 28 (second row) for each group. The broken lines represent 5 and 95% percentiles

**Fig. 3:**
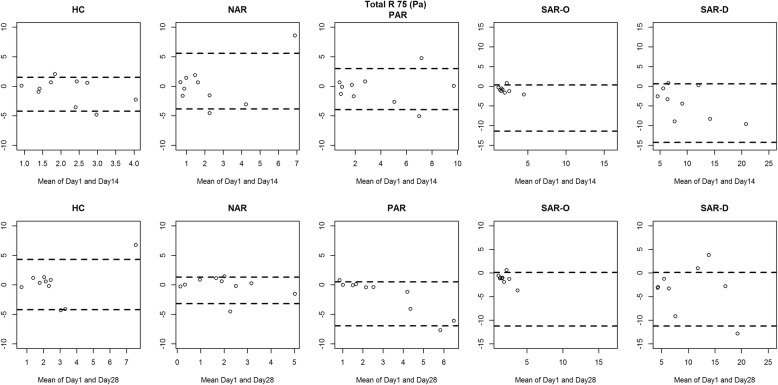
Bland-Altman plot: the difference between the Total R 75 (Pa) measurements of Day 1 and Day 14 (first row) and between Day 1 and Day 28 (second row) for each group. The broken lines represent 5 and 95% percentiles

**Fig. 4:**
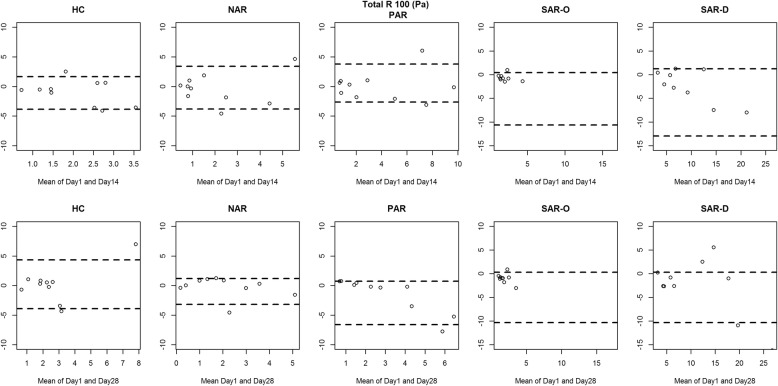
Bland-Altman plot: the difference between the Total R 100 (Pa) measurements of Day 1 and Day 14 (first row) and between Day 1 and Day 28 (second row) for each group. The broken lines represent 5 and 95% percentiles

**Fig. 5:**
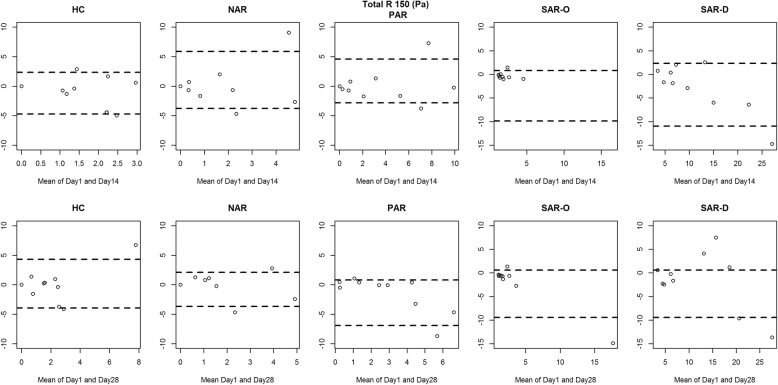
Bland-Altman plot: the difference between the Total R 150 (Pa) measurements of Day 1 and Day 14 (first row) and between Day 1 and Day 28 (second row) for each group. The broken lines represent 5 and 95% percentiles

Figures 2, 3, 4 and 5 show the between-day reproducibility of total combined R2, R 75 Pa, R 100 Pa and R 150 Pa, for each group of children. Specifically, the first row reports the reproducibility after 14 days from baseline (visit 2), and the second row reports the reproducibility after 28 days from baseline (visit 3). For all groups no evidence of heteroscedasticity was found, and therefore the statistical analysis was continued without logarithmic transformation. Point distribution appeared to be random, except for SAR-D, for which a decreasing trend was observed, and SAR-O, for which most of the measurements were clustered at small values.

Table 6 reports the CV and ICC values of Day 1 and Day 14 and between Day 1 and Day 28 by group. An unacceptable reproducibility was found since all CVs were ≥ 0.15 and most of the ICCs were not significant.

**Table 6:** CV and ICC between Day 1 and Day 14 (first column) and between Day 1 and Day 28 (second column) by group

	Day 14			Day 28		
	CV	ICC	*p*-value	CV	ICC	*p*-value
**R2**						
HC	0.395	0.004	0.719	0.429	0.228	0.461
NAR	0.525	0.475	0.171	0.366	0.646	**0.046**
PAR	0.352	0.71	**0.022**	0.256	0.306	0.362
SAR-O	0.337	0.55	0.106	0.341	0.681	**0.031**
SAR-D	0.201	0.781	**0.007**	0.239	0.776	**0.008**
**R 75 Pa**						
HC	0.481	−0.167	0.861	0.459	0.245	0.439
NAR	0.799	0.142	0.567	0.508	0.493	0.154
PAR	0.402	0.742	**0.014**	0.347	0.179	0.521
SAR-O	0.417	0.378	0.275	0.531	0.482	0.164
SAR-D	0.333	0.534	0.118	0.372	0.549	0.106
**R 100 Pa**						
HC	0.566	−0.239	0.903	0.469	0.308	0.36
NAR	0.663	0.316	0.35	0.529	0.531	0.121
PAR	0.465	0.769	**0.009**	0.398	0.262	0.417
SAR-O	0.356	0.437	0.21	0.459	0.551	0.105
SAR-D	0.23	0.681	**0.032**	0.255	0.708	**0.023**
**R 150 Pa**						
HC	0.865	−0.440	0.973	0.682	0.390	0.261
NAR	1.106	0.007	0.716	0.709	0.494	0.153
PAR	0.535	0.738	**0.015**	0.592	0.302	0.368
SAR-O	0.274	0.494	0.153	0.376	0.625	0.057
SAR-D	0.206	0.772	**0.008**	0.234	0.765	**0.010**

### Symptom data

Table 7 reports AUC and 95% CI for AAR parameters in predicting current symptoms of rhinitis in the overall study population. Of interest, in all the children reporting current symptoms of rhinitis a significant association with two items of T5SS, such sneezing and nasal obstruction, were found (*p* = 0.024 and *p* = 0.021, respectively).

**Table 7:** AUC and 95%CI for predicting current symptoms of rhinitis

	Current symptoms of rhinitis**		
R2	AUC	95% CI	
Total Inspiratory	**0.741**	**0.576**	**0.905**
Total Expiratory	0.689	0.495	0.884
Combined Total	**0.708**	**0.532**	**0.885**
**R75 Pa**			
Total Inspiratory	**0.822**	**0.699**	**0.945**
Total Expiratory	**0.746**	**0.57**	**0.922**
Combined Total	**0.792**	**0.639**	**0.944**
**R 100 Pa**			
Total Inspiratory	**0.75**	**0.591**	**0.909**
Total Expiratory	**0.769**	**0.627**	**0.911**
Combined Total	**0.773**	**0.619**	**0.927**
**R 150 Pa**			
Total Inspiratory	**0.701**	**0.536**	**0.865**
Total Expiratory	**0.735**	**0.604**	**0.866**
Combined Total	**0.723**	**0.572**	**0.875**

**ISAAC core question for rhinitis, n. 2: “In the past 12 months, has your child had a problem with sneezing, or a runny nose, or blocked nose when he/she did not have a cold or the flu? Yes/No″

## Discussion

In this paper, two common approaches used for assessing repeatability and reproducibility were compared; the focus was on the misleading results obtained when inappropriate tools are used. In fact, although the use of the CV has largely been discouraged, this warning appears to be still ignored among most clinicians.

A simulation study showed that ICC values estimated from data generated, assuming a given true CV, yielded moderate repeatability until CV was < 15%, while when data were generated from a mixed model, irrespective of the magnitude of the true ICC, CV reported conflicting results depending especially on the combination of mean and variance used for generating the data [34]. Indeed, when the mean value is close to zero, the coefficient of variation approaches infinity and is therefore sensitive to small changes in the mean. This is often the case if the values do not originate from a ratio scale. Repeatability and reproducibility should be assessed using a statistical test highlighting reliability of the measurement and not the differences between subjects.

The motivating dataset provided a good example of this; indeed, until now AAR repeatability has only been studied in adults [8–10]. Two studies reported repeatability in terms of CV, and only one reported both CV and ICC. CVs computed for our clinical data, are similar to other studies on healthy adults reporting unacceptable repeatability [9] and reproducibility [10]. However, when ICC is considered, our results suggest that AAR has good repeatability. Similarly, Silkoff et al. reported conflicting results depending on the statistical tool used: in particular good repeatability with ICC was observed (0.76, 0.70 and 0.96 for right, left and combined nasal resistance respectively), whereas, when CV was considered, unacceptable or poor repeatability was obtained for right and left nasal resistance (CV = 15.9% and CV = 12.9%) [8]. On the other hand, when ICC was used to assess reproducibility most of the ICCs were not significant. However, in order to test the null hypothesis of ICC ≤ 0.20, considering an expected ICC of at least 0.70 and two repeated measurements for subject with a 90% statistical power and a 5% significance level, a sample size of 21 subjects per group was needed [35]. Therefore, the Bland and Altman plot is preferred, given the powerful visual representation of the degree of agreement and the easy identification of bias, outliers, and any relationship between the variance in measures with the size of the mean [4]. Bland and Altman plots constructed for our clinical data showed no evidence of heteroscedasticity and point distribution appeared to be random, except for SAR-D and SAR-O. The difference in reproducibility between groups is unexplained; however, the required sample size to estimate reproducibility using the Bland-Altman plot setting an expected mean of differences 0.20, an expected standard deviation of differences of 0.10 and a maximum allowed difference between methods of 0.50, was of 26 subjects [22]. Therefore, since the AAR repeatability in children with upper airway obstructive symptoms has not been investigated before, larger numbers of cases and more repeated measurement in prospective are needed to better determine reproducibility.

The present paper might suggest that, due to the use of inappropriate statistical tools, AAR repeatability and reproducibility may have been underestimated in previous assessments. Overall, our results highlight the clinical reliability of AAR both in healthy children and in ones with rhinitis. Furthermore, we showed good performance of AAR parameters in predicting current symptoms of rhinitis in the overall study population. This suggests that a more accurate reproducible measurement well correlates with patient’s symptoms, highlighting the additional value of AAR performance in clinical practice.

## Conclusions

Physicians dealing with clinical data should carefully choose the most suitable statistical tools for assessing repeatability and reproducibility. The results of the present study support the clinical reliability of AAR parameters that showed good repeatability both in healthy and in rhinitis children.

## Data Availability

All data and materials are available upon request.

## Abbreviations

AAR: Anterior Active Rhinomanometry
ICC: Intraclass Correlation Coefficient
CV: Coefficient of Variation
HC: Healthy Children
NAR: Non-allergic rhinitis
PAR: Perennial allergic rhinitis
SAR-D: Seasonal allergic rhinitis during the pollen season
SAR-O: Seasonal allergic rhinitis outside the pollen season
T5SS: Total Five Symptoms Score
